# Risk Factors for Catheter-Related Thrombosis

**DOI:** 10.3390/jcm15103932

**Published:** 2026-05-20

**Authors:** Leyla La Cava, Davide Giustivi, Arianna Bartoli, Alessia Meschia, Federica Cirigliano, Teresa Lanzi, Beatrice Tramalloni, Maria Calloni, Paolo Zappa, Alba Taino, Giacomo Ronzoni, Antonella Foschi, Igor Giarretta, Marco Gemma, Adam Fabiani, Chiara Cogliati, Antonio Gidaro

**Affiliations:** 1 Internal Medicine, Ospedale Luigi Sacco, Aziende Socio Sanitarie Territoriali (ASST) Fatebenefratelli-Sacco, 20157 Milan, Italy; lacava.leyla@asst-fbf-sacco.it (L.L.C.); bartoli.arianna@asst-fbf-sacco.it (A.B.); meschia.alessia@asst-fbf-sacco.it (A.M.); cirigliano.federica@asst-fbf-sacco.it (F.C.); lanzi.teresa@asst-fbf-sacco.it (T.L.); tramalloni.beatrice@asst-fbf-sacco.it (B.T.); calloni.maria@asst-fbf-sacco.it (M.C.); zappa.paolo@asst-fbf-sacco.it (P.Z.); taino.alba@asst-fbf-sacco.it (A.T.); giacomo.ronzoni@gmail.com (G.R.); cogliati.chiara@asst-fbf-sacco.it (C.C.); 2 Vascular Access Team, Post-Anesthesia Care Unit, Aziende Socio Sanitarie Territoriali (ASST) Lodi, 26900 Lodi, Italy; davide.giustivi@asst-lodi.it; 3 Unit II, Department of Infectious Diseases, L. Sacco Hospital, Aziende Socio Sanitarie Territoriali (ASST) Fatebenefratelli-Sacco, 20157 Milan, Italy; foschi.antonella@asst-fbf-sacco.it; 4 Department of Internal Medicine, Ospedale di Circolo e Fondazione Macchi, 21100 Varese, Italy; igor.giarretta@asst-settelaghi.it; 5 Intensive Care Unit, Department of Neurosurgery, Fondazione IRCCS Istituto Neurologico Carlo Besta, 20133 Milan, Italy; marco.gemma@istituto-besta.it; 6 Vascular Access Team, Cardiac Surgery Intensive Care Unit, Cardiothoracic-Vascular Department, Azienda Sanitaria Universitaria Giuliano-Isontina, 34125 Trieste, Italy; adam.fabiani@units.it; 7 Department of Biomedical and Clinical Sciences, Università degli Studi di Milano, 20157 Milan, Italy

**Keywords:** catheter-related thrombosis, central venous catheter, peripherally inserted central catheters, midline catheters, anticoagulants, platelet aggregation inhibitors, thromboprophylaxis, compression ultrasonography

## Abstract

**Background:** Although guidelines emphasize proper insertion techniques and tip positioning, catheter-related thrombosis (CRT) remains a common and clinically significant complication of peripherally inserted central catheters (PICCs) and midline catheters (MCs). In this context, the use of pharmacological prophylaxis is still debated. This study aims to assess the incidence of CRT in patients receiving anticoagulant therapy (therapeutic or prophylactic) and antiplatelet therapy. **Methods:** This retrospective study was conducted at a tertiary care hospital and included adult patients from March 2021 to May 2023. Six potential confounders were analyzed: anticoagulation status (none, prophylaxis, therapeutic), antiplatelet therapy, tip position (PICCs vs. MCs), number of lumens, CRT risk factors, and drug infusion requiring central access. CRT was diagnosed in symptomatic patients using compression ultrasonography. Propensity score weighting and logistic regression were employed to estimate odds ratios (OR) and average treatment effects. **Results:** A total of 1431 patients were enrolled. PICCs and therapeutic anticoagulant therapy were highly protective against CRT (OR 0.068 [95% CI 0.013–0.2] and OR 0.007 [95% CI 0.001–0.046], respectively). Prophylactic anticoagulant therapy (OR 0.328 [95% CI 0.200–0.519]) and antiplatelet therapy (OR 0.342 [95% CI 0.182–0.595]) also showed protective effects. At the same time, neither the number of lumens, the presence of risk factors, nor the infusion of irritating drugs was independently associated with CRT. **Conclusions:** The use of anticoagulant drugs (both prophylactic and therapeutic), antiplatelet therapy, and PICC use significantly lowered the risk of CRT. The findings support personalized prevention strategies and underscore the need for a well-designed randomized controlled trial to validate these findings.

## 1. Introduction

Catheter-related thrombosis (CRT) is a prevalent and potentially severe complication associated with peripherally inserted central catheters (PICCs) and midline catheters (MCs) [1]. CRT may result in the interruption or failure of intravenous therapies, necessitating catheter removal or replacement, and may escalate treatment to include systemic anticoagulation. In severe instances, CRT can lead to pulmonary embolism, infection, and long-term venous damage [2]. Consequently, CRT imposes a substantial clinical and economic burden, particularly within high-risk populations.

The pathophysiology of CRT is intricate and multifactorial and can be conceptualized through the framework of Virchow’s triad: endothelial injury, alterations in venous blood flow (stasis), and a hypercoagulable state [3]. A confluence of device-related, procedural, and patient-specific factors influences these mechanisms. Device-related contributors include catheter diameter, number of lumens, surface material, and the presence or absence of adequate stabilization, all of which can affect shear stress and endothelial trauma. Procedural variables, including the insertion technique, number of puncture attempts, and final tip location, may further predispose to endothelial disruption, vessel inflammation, and localized blood stasis. Meanwhile, patient-related conditions, such as active malignancy, systemic infection, renal impairment, inherited or acquired thrombophilia, and a history of venous thromboembolism, exacerbate the prothrombotic environment, thereby increasing the risk of CRT development [4,5].

International guidelines recommend minimizing the catheter-to-vein ratio (CVR), discouraging the use of dual-lumen catheters without a clinical indication, ensuring proper catheter stabilization, and adhering strictly to standardized insertion protocols to promote safe and effective vascular access [6,7]. Notwithstanding these preventive strategies, the optimal choice between PICCs and MCs remains a topic of ongoing debate, particularly within populations at increased risk of thrombotic complications.

Current recommendations regarding pharmacologic prophylaxis are more conservative [8]. International guidelines discourage routine pharmacologic thromboprophylaxis solely to prevent CRT, primarily because of conflicting evidence from prior studies [7]. Some trials have suggested that anticoagulant prophylaxis may reduce CRT rates, while others have shown no significant benefit, particularly in patients without cancer or other major risk factors [9,10,11].

Another contentious aspect concerns catheter tip positioning [12]. In PICCs, the catheter tip is positioned at the cavoatrial junction or in the proximal third of the superior vena cava, a position generally considered optimal for rapid drug hemodilution and for minimizing endothelial damage. In contrast, in MCs, the tip’s placement remains debated, with three possible positions recognized: distal to the axillary fold, within the midaxillary vein (the thoracic segment of the axillary vein), or within the subclavian vein. However, some studies suggest that the risk of CRT may not differ significantly between centrally positioned tips and those terminating more peripherally, such as in MCs [13,14]. This uncertainty is especially relevant when weighing procedural complexity, patient tolerance, and healthcare system costs.

Given the ongoing debate in the literature and the heterogeneity of clinical practice, additional research is essential to elucidate the protective effects of antithrombotic interventions and tip positioning on the incidence of CRT. Retrospective analyses of large cohorts, although constrained by inherent biases, can yield valuable insights into real-world outcomes, particularly when adjusted for known confounders.

This retrospective cohort study aims to assess the incidence of CRT in patients undergoing PICC or MC implantation and to evaluate the possible protective effect on CRT of antithrombotic prophylaxis, anticoagulation, and antiplatelet therapy, accounting for several potentially significant covariates such as catheter tip position, number of lumens, infusion of medications necessitating central venous access, and CRT risk factors (solid or hematologic active cancer, recent surgery, bedridden, heart failure, liver failure, chronic kidney disease, chronic inflammatory bowel diseases, obesity, inherited or acquired thrombophilia). Through this analysis, the authors seek to provide practical insights to inform evidence-based vascular access management.

## 2. Materials and Methods

### 2.1. Study Design

This retrospective study was conducted at Luigi Sacco Hospital in Milan, a large university hospital, in accordance with the STROBE statement [15]. The Luigi Sacco Hospital has had an established vascular access team (VAT) since 2018, which has provided standardized procedures and data reporting. Data were extracted from the local database, and, when information was missing, the medical records were reviewed. The observation period ran from 1 March 2021 to 31 May 2023.

### 2.2. Population

All patients aged 18 years or older who underwent a PICC or MC implantation were enrolled, regardless of their entry diagnosis. Exclusion criteria included: age under 18 years; admission to an intensive care unit; and the presence of more than one catheter during hospitalization (i.e., venous access in situ before the start of the study).

### 2.3. Ethics

All subjects provided their written informed consent for inclusion. This study was conducted in accordance with the Declaration of Helsinki, and the protocol was approved by the Luigi Sacco Hospital Institutional Review Board (Research Ethics Committee approval number 30236/2024).

### 2.4. Outcome Definitions

All patients enrolled before catheter removal were systematically examined for signs of thrombosis. Each catheter was removed only after thrombotic symptoms were ruled out. Symptomatic patients with suspected CRT (venous distension, limb swelling with or without pain) during dwell time or upon catheter removal were evaluated to exclude CRT from the VAT.

### 2.5. Diagnostic Criteria for Catheter-Related Thrombosis

**Figure 1:**
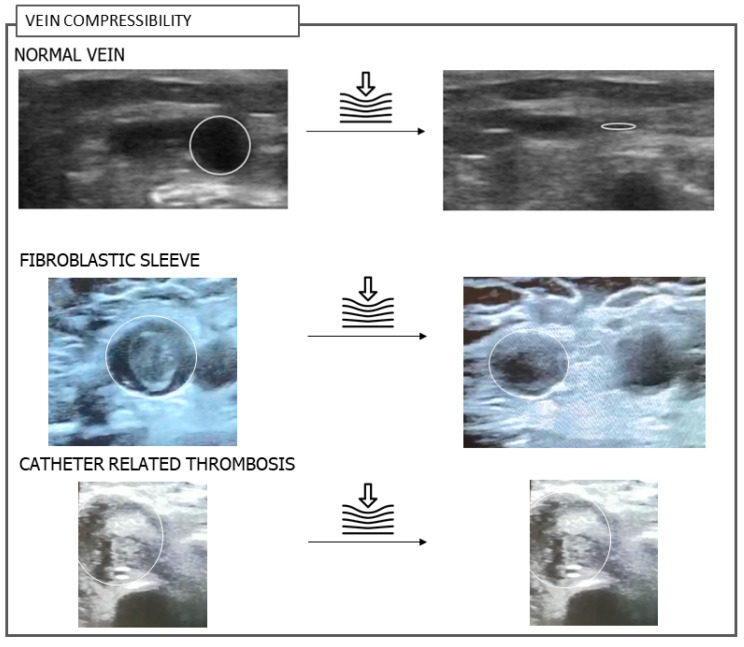
Ultrasonographic differentiation among normal vein, fibroblastic sleeve, and catheter-related thrombosis. B-mode ultrasound images illustrating: a normal vein with full compressibility; a fibroblastic sleeve, showing a partially compressible lumen with a hyperechoic layer surrounding the catheter but no vessel wall involvement; and catheter-related thrombosis, characterized by a non-compressible vein and intraluminal echogenic material. These examples demonstrate the key sonographic criteria for distinguishing CRT from benign pericatheter changes.

The CRT diagnosis was made using compression ultrasound (CUS). During site scanning, the vein was examined in a short-axis view, and catheter images were obtained in an out-of-plane B-mode-only view. The probe was utilized to compress the vein and assess thrombosis by its complete non-compressibility [16]. On the other hand, CRT was excluded when a fibroblastic sleeve was identified, as evidenced by a partially compressible vein and a hyperechoic layer around the catheter, without involvement of the vessel wall [17]. When uncertain, the Color-Doppler ring sign was used to differentiate between CRT and a fibroblastic sleeve (Figure 1).

### 2.6. Catheter Insertion, Management, and Diagnosis of CRT

In this study, 4 Fr MC, 25 cm long, single-lumen, 4 Fr PICC, 55 cm long, single-lumen, or 5 Fr PICC, 55 cm long, double-lumen, were used (Numantec, 46019 Viadana, Italy). All devices were non-valved polyurethane and were inserted by VAT personnel using the “Safe Insertion of PICC 2.0” protocol after a preprocedural ultrasound assessment using the RaPeVA (Rapid Peripheral Venous Assessment) to exclude coexisting thrombosis and evaluate the venous system, with a catheter-to-vein ratio of at least 1:3 [18]. Correct tip location was confirmed by an ECG-guided technique when feasible or by ultrasound. Proper MC tip placement in the axillary and/or subclavian vein was confirmed by ultrasound of the subclavicular area during insertion.

Ward nurses performed catheter management in accordance with local protocols (flushing with saline solution before and after use and replacing the dressing every 7 days or sooner if soiled or detached).

### 2.7. Cohort Definition and Grouping Criteria

After a thorough review of the literature, six variables were identified that could affect CRT as either risk or protective factors. Five of these variables were dichotomous. These include antiplatelet use (no/yes) [19], central catheter tip location (PICC/MC) [2], number of lumens (one/two) [2], use of drug-requiring central catheters—such as solutions with a pH < 5 or >9, osmolarity > 600 mOsm/L and a reported incidence of thrombophlebitis (≥5%) when infused in peripheral veins or with vesicant properties (no/yes)—and the presence of at least one CRT risk factor (no/yes): active solid or hematologic cancer, recent surgery, bedridden status, heart failure, liver failure, chronic kidney disease, chronic inflammatory bowel diseases, obesity, or inherited or acquired thrombophilia [2,20,21,22,23]. The sixth variable, anticoagulation therapy, was categorized into three groups [7,8,9,10,11]:

1. No Anticoagulant or Prophylaxis Therapy: This group includes patients who are not receiving any form of anticoagulant or prophylactic treatment.
2. Prophylaxis During Hospitalization: This group consists of individuals at high risk of venous thrombosis, as determined by their physician before catheter insertion, or those who have been on long-term prophylaxis for previous venous thrombosis (more than six months since the event). Patients receiving thromboprophylaxis therapy were defined as follows:
  - Low-Molecular-Weight Heparin (LMWH): Enoxaparin 4000 UI if the patient’s weight is under 100 kg; Enoxaparin 6000 UI if the weight is over 100 kg.
  - Fondaparinux 2.5 mg or 1.5 if renal failure with GFR under 30 mL/min
  - Direct Anticoagulants (DOAC): After six months from the initial venous thrombosis event, patients on reduced-dose DOAC as secondary prevention for venous thromboembolism (VTE), such as apixaban (2.5 mg twice a day) or rivaroxaban (10 mg).
3. Full Dosage Anticoagulation: This group includes patients with specific clinical conditions such as atrial fibrillation, VTE (within the first six months of treatment or extended therapy at full dosage), and cardiac valve prostheses. The anticoagulation therapy for these patients is defined as follows:
  - LMWH: 100 UI per kg, administered twice daily.
  - Fondaparinux 7.5 mg or 10 mg is for individuals weighing over 100 kg
  - DOAC or Vitamin K Antagonist (VKA): Given at the appropriate dosage for atrial fibrillation or VTE during the first six months.

### 2.8. Sample Size Calculation

A CRT incidence of 2% was assumed for patients without risk factors and 4% for those with risk factors, with a sensitivity of 80% (α = 0.05, power = 80%). Thus, a sample size of at least 1141 patients was estimated.

### 2.9. Statistical Analysis

Descriptive statistics were used to summarize patient and catheter characteristics. Continuous variables were assessed for normality using the Shapiro–Wilk test and reported as mean ± standard deviation (SD) or median (IQR), as appropriate. Categorical variables were expressed as a number (percentage).

We estimated risk differences (RD) and 95% confidence intervals using the Newcombe method based on Wilson score intervals for observed counts. When subgroup counts were unavailable (e.g., for prophylaxis), RD was derived from odds ratios adjusted for the baseline risk in the reference group. A generalized linear model (GLM) was used to generate propensity score weights for balancing, with the Average Treatment Effect (ATE) as the estimand. Anticoagulation was kept as the treatment variable, and the five aforementioned dichotomous potential confounders were taken as covariates. Covariate balance before and after adjusting was graphically evaluated with “Love” plots.

Thereafter, a logistic model was built using the calculated propensity score weights. Given the issue of rare events and the possibility of complete separation, the penalized likelihood method originally proposed by Firth and fully described by Heinze was applied (in the R package logistf, Version 4.5.3 released on 11 March 2026). Variables with *p* < 0.20 were considered during the variable selection process. Odds Ratios (OR) are reported along with their 95% Confidence Intervals (CIs).

The statistical analysis was performed using the latest version of R (R Core Team, 2025, Version 4.5.3 released on 11 March 2026). _R: A Language and Environment for Statistical Computing_. R Foundation for Statistical Computing, Vienna, Austria. https://www.R-project.org/, Version 4.5.3 released on 11 March 2026.

### 2.10. AI Statement

Generative AI (Grammarly, v.1.161.0.0 relased in April 2026) was used only during the final proofreading phase of this paper’s preparation. The usage was limited to flag potentially unclear sections for the authors’ manual revision. The named authors performed all drafting, analysis, and revisions. The final version of the manuscript was reviewed and approved by all authors.

## 3. Results

The study enrolled 1431 patients, and data on the enrolled population are shown in Table 1.

The median age of participants was 78 years, with 669 (46.8%) being male. Mean catheter dwell time was 11 (6–21) days for the whole population, 12 (8–25) days for CRT; no statistical difference was found comparing patients with and without CRT for catheter dwell time (*p* = 0.31). During catheter insertion, 358 (25%) patients required a PICC, and the remaining 1073 (75%) required an MC. A significant proportion of patients (1024; 72%) received drugs that would have required central catheter use during the device’s dwell time. This resulted in 666 MCs (46.5% of the whole population) being utilized to infuse irritant medications, due to a change in drug prescription during hospitalization after MC insertion. Additionally, 1084 patients (76%) had risk factors for CRT. Regarding treatment, 761 (53.2%) received prophylactic anticoagulant therapy, while 349 (24.4%) received therapeutic anticoagulant therapy. Enoxaparin was the most used anticoagulant (885; 79.7%), followed by Apixaban (64; 5.8%) and Fondaparinux (54; 4.9%) (Table 2).

In terms of antiplatelet therapy, Cardioaspirin was the most common (305; 73.8%), followed by Lysine acetylsalicylate (67; 16.8%) and Clopidogrel (36; 8.7%).

**Figure 2:**
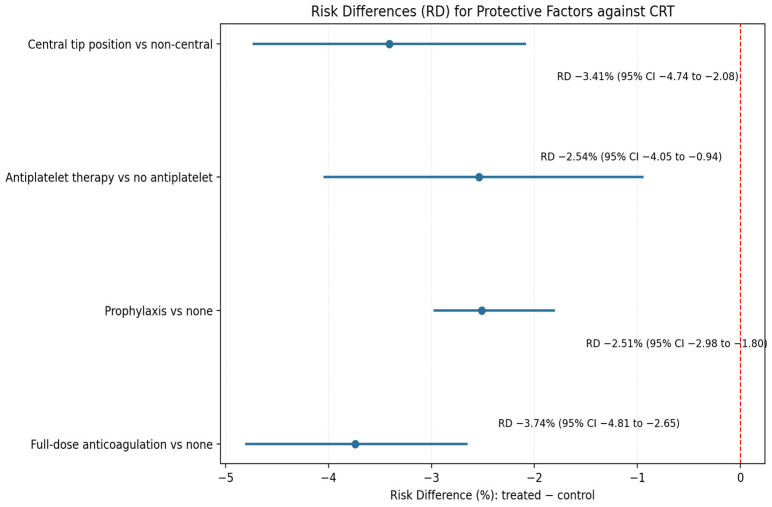
Forest plot showing absolute risk differences (RD) with 95% confidence intervals for protective factors against catheter-related thrombosis (CRT). Negative values indicate a lower CRT risk than in the reference group. Full-dose anticoagulation (RD −3.74%, 95% CI −4.81 to −2.65) and central tip position (RD −3.41%, 95% CI −4.74 to −2.08) provided the greatest absolute risk reduction, followed by prophylactic anticoagulation (RD −2.51%, 95% CI −2.98 to −1.80) and antiplatelet therapy (RD −2.54%, 95% CI −4.05 to −0.94).

During the study, 25 CRTs were found, with an incidence of 1.6/1000 catheter days; all were in the deep veins, and no superficial ones occurred. CRT was more common in patients who did not receive anticoagulant therapy (12 [48%]) and in those with CRT risk factors or who were treated with solutions with a pH < 5 or >9, osmolarity > 600 mOsm/L, and a reported incidence of thrombophlebitis if infused in peripheral veins ≥ 5% or with vesicant properties (18 [both 72%]). Notably, no cases of CRT were reported among patients receiving therapeutic anticoagulation. The RD for full-dose anticoagulation versus no anticoagulation was −3.74% (95% CI, −4.81 to −2.65). Prophylaxis reduced risk by −2.51% (95% CI −2.98 to −1.80), antiplatelet therapy by −2.54% (95% CI −4.05 to −0.94), and central tip position by −3.41% (95% CI −4.74 to −2.08), Figure 2.

In a multivariate propensity score-weighted model, anticoagulant therapy effectively protected against CRT development: with respect to “no anticoagulation”, prophylactic anticoagulation exhibited *p* < 0.001, OR 0.328 [95% CI 0.200–0.519], and full anticoagulation *p* < 0.001, OR 0.007 [95% CI 0.001–0.046]. In the same model, antiplatelet therapy (*p*-value < 0.001; OR 0.342 [95% CI 0.182–0.595]) and central tip position (*p*-value < 0.001; OR 0.068 [95% CI 0.013–0.2]) were protective against CRT development. No significant effect on CRT development was observed with respect to lumen number, the presence of CRT risk factors, or the use of drugs that require central catheters.

Observed values for statistically significant confounders are shown in Figure 2.

## 4. Discussion

In this study, the risk of CRT was significantly lower among patients receiving (i) pharmacologic thromboprophylaxis, (ii) full-dose anticoagulation, (iii) concurrent antiplatelet therapy, and (iv) devices with a central (cavoatrial junction/superior vena cava) tip position, such as PICCs, after applying inverse probability weighting for measured confounders. Expressing results as risk differences emphasizes the absolute benefit of interventions. Full-dose anticoagulation primarily protects through CRT (RD −3.74%), whereas prophylaxis, antiplatelet therapy, and central tip positioning reduce risk by 2–3 percentage points, yielding meaningful reductions per 100 patients treated.

In contrast, the number of lumens, the presence of pre-specified CRT risk factors, and administration of drugs typically requiring central access were not independently associated with CRT. These findings reinforce the multifactorial nature of CRT and the importance of modifiable, procedure-related factors (especially tip location), while also suggesting a role for selective pharmacologic strategies in appropriately risk-stratified patients [2].

This study highlights the protective effect of therapeutic anticoagulation in preventing CRT. Across cohorts, no CRT events were observed among patients receiving therapeutic anticoagulation, consistent with current guidelines and recent studies that consider anticoagulation a key component of CRT management, particularly when the catheter remains functional and necessary [24,25]. Guidelines recommend that, once CRT is confirmed, anticoagulation should be administered for a set period and continued as long as the catheter remains in place, provided the bleeding risk is acceptable [24,25]. This protective effect of anticoagulation is further supported by a recent study by Cavallaro et al., which reported a recurrence rate of only 0.5 events per 100 person-years in a cancer population receiving anticoagulation following prior CRT [26]. This is equivalent to 0.001% per day, which is quite similar to our cohort’s estimated risk, OR 0.007 [95% CI 0.001–0.046].

Routine antithrombotic prophylaxis is not recommended in patients with PICCs or MCs due to inconsistent evidence and bleeding risk concerns, particularly in unselected populations [9,10,24]. The 2018 Cochrane review in oncology patients with central venous catheters (CVCs) concluded that anticoagulant prophylaxis did not consistently reduce symptomatic CRT and was associated with bleeding, supporting the prevailing conservative stance [10]. Similarly, a randomized controlled trial (RCT) phase III in cancer patients showed no clear advantage of low-dose warfarin or LMWH over control for CRT prevention [9]. Moreover, a pilot RCT evaluating rivaroxaban (10 mg daily) found no significant difference in preventing venous thromboembolism [27]. By contrast, this real-world analysis suggests that selective prophylaxis, as implemented in routine care at the study site due to comorbidities and bedridden status during hospitalization, was associated with a substantially lower risk of CRT than no prophylaxis. This finding confirms a recent meta-analysis showing that prophylaxis with LMWH, VKA, or DOACs reduces the overall incidence of CRT compared with no prophylaxis, without increasing the risk of major bleeding. However, minor bleeding was more frequent [28]. Notably, the AVERT trial showed that apixaban (2.5 mg twice daily) significantly reduced the risk of CRT in ambulatory cancer patients with CVCs (4.8% vs. 18.7% with placebo), without excess major bleeding [29]. In addition, novel approaches, such as factor XI inhibition with gruticibart, demonstrated promising results in reducing CRT on surveillance ultrasound without safety concerns [30]. All the evidence, considered together, supports individualized, risk-based decisions rather than blanket prophylaxis. The findings of this study support a targeted strategy for patients with multiple converging CRT risk factors who have acceptable bleeding profiles [24,31]. Prospective RCT data in mixed medical populations (non-oncology and oncology) remain a key unmet need to confirm this signal [10].

Antiplatelet use was associated with a lower risk of CRT in this cohort. Evidence in this domain is limited, but an observational oncology study suggested that aspirin prophylaxis may reduce PICC-related thrombosis and has an acceptable safety profile [19]. Neither major guidelines nor systematic reviews currently recommend antiplatelet agents solely for CRT prevention, reflecting the paucity of high-quality RCT data [2,24]. Nonetheless, in patients already receiving antiplatelet therapy for cardiovascular indications, the findings of this study and prior reports suggest that antiplatelet therapy may confer additional protection against CRT. This area warrants pragmatic trials [2,19,24].

The study observed a strong protective effect for central tip position (cavoatrial junction/superior vena cava) in PICCs. This is biologically plausible because central tips minimize endothelial contact, turbulence, and stasis, and align with infusion therapy standards and contemporary studies [6,32,33]. The CRT rate reported in this study is lower than that reported by Bentridi et al. in a randomized controlled trial comparing Midline and PICC, at 5.2% and 4.4%, respectively [34]. The two studies differ in tip-location methods: Bentridi et al. used blind insertion for MC and fluoroscopy for PICC [34]; this cohort used ultrasound for MC and Intracavitary ECG for PICC. ECG-guided positioning improves first-pass accuracy, reduces malposition, and has been linked to favorable thrombotic outcomes compared with traditional methods [6,32,33]. Collectively, these data support the use of standardized PICC placement to reduce CRT incidence, as recommended by practice standards [6,32,33].

Prior studies have linked larger-diameter catheters, such as multilumen catheters, to an increased risk of CRT, especially in oncology settings [2]. In this analysis, lumens were not independently associated with CRT after adjustment for confounders. This may be due to the consistent use of insertion bundles, which ensure proper stabilization, and strict adherence to a favorable Catheter-to-Vein Ratio (i.e., 1:3) [2,6]. Maintaining an appropriate Catheter-to-Vein Ratio reduces the risk of CRT associated with larger catheters, such as multilumen catheters.

In our population, a large majority of patients (1024; 72%) received medications that required central catheter use during the devices’ dwell time. As a result, in almost half of the entire patient population, MCs were used for the infusion of irritant medications due to a change in drug prescription. Despite this, administering an irritant drug via a peripheral venous catheter was not identified as a risk factor for CRT. These findings contrast with recent literature addressing drug characteristics as a safe concern [20,21,22,23]. The likely reason for this discrepancy is that this classification applies to short peripheral catheters, for which evidence indicates that their use is associated with catheter failure, fibroblastic sleeve formation, and thrombosis [35]. The same can also be confirmed for long peripheral catheters (LPCs), as shown in the randomized controlled trial by Scarano et al., in which vancomycin was infused through LPCs [36]. Still, the experimentation was terminated after 14 patients due to the high risk of fibroblastic sleeves and thrombosis [36]. Conversely, recent evidence from Zhang et al. supports the safety of administering irritant medications through MCs when the tip is positioned in the thoracic tract of the axillary or subclavian vein. In their extensive retrospective cohort study of 1613 patients, no significant link was found between irritant infusion and catheter-related complications, even after propensity score matching and sensitivity analyses [37]. According to Zhang et al., a systematic review about MCs and LPCs showed that the catheter tip’s location in the axillary vein in the thoracic tract reduced overall complications, supporting the safety of more central terminal locations within the upper limb venous pathway when appropriately indicated [38]. Based on the HERITAGE study results, it is now established that the longer the catheter, the lower the risk of complications [39], likely because the catheter-to-vein ratio at the tip level, which is lower in more proximal veins, is the most critical factor [40].

Considering that infused drug characteristics did not influence CRT risk and that the protective effect of the central tip catheter (OR 0.068, 95% CI 0.013–0.2) is comparable to that of therapeutic anticoagulation (OR 0.007, 95% CI 0.001–0.046), this finding suggests that MCs may be a viable alternative for patients on anticoagulation therapy. However, while these results challenge the current consensus recommendations favoring central access for the administration of irritants, it is important to stress that these statements should be interpreted with extreme caution, as they apply exclusively to MCs with tips located in veins within the thoracic tract and to patients receiving therapeutic doses of anticoagulants. The hypothesis that, in this population, midline catheters can be used effectively and safely as an alternative to central-tip catheters—at least for short-term use—is particularly compelling, given that midline catheters are associated with lower material costs and shorter insertion times. These results emphasize the need for RCTs to confirm the viability of MC use in this context.

Additionally, the use of anticoagulation and prophylaxis during hospitalization could be a confounding factor, which may explain why extensive systematic reviews have shown that MCs have similar or lower CRT rates than PICCs [41].

### Strengths and Limitations

Strengths include a significant real-world cohort, a standardized vascular access protocol with a formal tip strategy, and analytic adjustment using propensity score weighting. Consistent with guideline-informed practice, B-mode ultrasonography was used as the primary diagnostic modality for symptomatic upper-extremity thrombosis; non-compressibility and direct visualization around the catheter remain the cornerstones of CRT diagnosis [16]. Differentiating fibroblastic (fibrin) sleeves from true intraluminal or mural thrombosis is critical, because sleeves may be partially compressible and do not invariably require systemic anticoagulation; recent correspondence underscores the importance of strict ultrasonographic criteria to avoid overdiagnosis and overtreatment [17]. The CRT evaluation strategy was guided by these principles, which may partly explain the relatively low overall rate observed. The heterogeneity of drugs used for prophylaxis and full anticoagulation therapy is both a limitation and a strength, as it offers a window into the real world. Limitations include the retrospective design, potential residual confounding, lack of systematic ultrasound screening (only symptomatic CRTs were captured), heterogeneity in antithrombotic agents/doses, and limited detail on bleeding outcomes. External validity may be influenced by local practice patterns (e.g., high penetration of ECG-guided tip confirmation and insertion bundles) [6,32,33]. Last but not least, bleeding events were not systematically collected; this remains a relevant limitation, particularly when interpreting the apparent protective effect of anticoagulation. Therefore, our findings should not be interpreted as an endorsement of routine prophylaxis.

## 5. Conclusions

In a large real-world cohort, therapeutic anticoagulation, selective thromboprophylaxis, concomitant antiplatelet therapy, and central tip positioning were each associated with lower CRT risk, whereas lumen number and drug-class requirements showed no independent effect after adjustment. This study provides valuable insights for healthcare professionals, helping them determine the most appropriate catheterization method based on patients’ CRT risk factors. Further research is needed to establish the efficacy of anticoagulation therapy and prophylaxis in preventing CRT. Until then, clinicians must weigh the potential benefits and risks of these interventions on a case-by-case basis, considering each patient’s medical history, comorbidities, and other factors that may affect their risk of thrombosis.

## Figures and Tables

**Table 1:** Summary of Population and Clinical Characteristics.

	Total (n; %)	CRT (n; %)
	1431	25 (1.7%)
Age, median (IQR)	78 (64–85)	75 (57–86)
Male	669 (46.8%)	5 (20%)
Female	762 (53.2%)	20 (80%)
Time from hospital admission to catheter insertion (IQR) days	3 (1–8)	3 (1–6)
Reason for catheter insertion		
Difficult intravenous venous access with intravenous therapy	1111 (77.6%)	21 (84%)
Parenteral nutrition	176 (12.3%)	4 (16%)
Chemotherapy	66 (4.6%)	0
Surgery	42 (2.9%)	0
Transfusion	36 (2.5%)	0
Catheter’s dwell time (IQR) days	11 (6–21)	12 (8–25)
Infusion of a drug requiring a central tip catheter	1024 (72%)	18 (72%)
Infusion of a drug compatible with a peripheral tip catheter	407 (28%)	7 (28%)
PICC	358 (25%)	1 (4%)
Midline	1073 (75%)	24 (96%)
Catheter with 1 lumen	1235 (86%)	24 (96%)
Catheter with more than 1 lumen	196 (14%)	1 (4%)
Patients with CRT risk factors	1084 (76%)	18 (72%)
Patients without CRT risk factors	347 (24%)	7 (28%)
Antiplatelet therapy	413 (28.9%)	5 (20%)
No antiplatelet therapy	1018 (71.1%)	20 (80%)
No Anticoagulant Therapy	321 (22.1%)	12 (48%)
Prophylaxis	761 (53.2%)	13 (48%)
Anticoagulant Therapy	349 (24.4%)	0 (0%)

**Table 2:** Anticoagulant and Antiplatelet Therapy.

Anticoagulant Therapy			
Drug	Prophylactic	Therapeutic	Total (N/%)
Enoxaparin	683 (89.8%)	202 (57.8%)	885 (79.7%)
Apixaban	20 (2.6%)	44 (12.6%)	64 (5.8%)
Edoxaban	0	41 (11.9%)	41 (3.7%)
Rivaroxaban	13 (1.7%)	23 (6.6%)	36 (3.2%)
Fondaparinux	45 (5.9%)	9 (2.6%)	54 (4.9%)
Warfarin		26 (7.4%)	26 (2.3%)
Dabigatran		4 (1.1%)	4 (0.4%)
**Antiplatelet Therapy**			
**Drug**	**Total (N/%)**		
Cardioaspirin	305 (73.8%)		
Lysineacetylsalicylate	67 (16.2%)		
Clopidogrel	36 (8.7%)		
Prasugrel	—		
Cardioaspirin + Prasugrel	1 (0.3%)		
Cardioaspirin + Clopidogrel	4 (1%)		

## Data Availability

The study data will be made available upon request to the corresponding author.

## Abbreviations

The following abbreviations are used in this manuscript:

CRT	Catheter-Related Thrombosis
PICC	Peripherally Inserted Central Catheter
MC	Midline Catheter
CVC	Central Venous Catheter
LPC	Long Peripheral Catheter
LMWH	Low-Molecular-Weight Heparin
DOAC	Direct Oral Anticoagulants
VKA	Vitamin K Antagonist
VTE	Venous Thromboembolism
OR	Odds Ratio
CI	Confidence Interval
RD	Risk Difference
GLM	Generalized Linear Model
ATE	Average Treatment Effect
SD	Standard Deviation
IQR	Interquartile Range
CUS	Compression Ultrasonography
ECG	Electrocardiogram
VAT	Vascular Access Team
STROBE	Strengthening the Reporting of Observational Studies in Epidemiology
RCT	Randomized Controlled Trial
IRCCS	Istituto di Ricovero e Cura a Carattere Scientifico
CVR	Catheter-to-Vein Ratio
GFR	Glomerular Filtration Rate
Fr	French gauge
AI	Artificial Intelligence
